# A commentary on “Prognostic value of iron-metabolism biomarkers in critically ill patients with atrial fibrillation: a machine learning-based retrospective cohort study”

**DOI:** 10.1097/JS9.0000000000002978

**Published:** 2025-07-08

**Authors:** Xuesi Chen, Xiaoya Mao, Minmin Cai

**Affiliations:** a Department of Cardiology, the Affiliated People’s Hospital of Ningbo University, Ningbo, Zhejiang, China; b Department of Anesthesiology, the Affiliated People’s Hospital of Ningbo University, Ningbo, Zhejiang, China

*Dear Editor,*

We read with considerable interest the article by Huang *et al* on the “Prognostic value of iron-metabolism biomarkers in critically ill patients with atrial fibrillation: a machine learning-based retrospective cohort study”[1]. The authors provide compelling evidence that iron-metabolism indicators, particularly total iron-binding capacity, serve as significant prognostic factors for one-year mortality in critically ill patients with atrial fibrillation (AF). While these are valuable insights, we believe several aspects warrant further discussion. This study is compliant with the TITAN Guidelines 2025 – governing declaration and use of AI[2].

Firstly, the authors appropriately acknowledge the unclear causal relationship between iron dysregulation and outcomes. However, recent Mendelian randomization studies offer compelling evidence for a causal link. Genetically determined higher iron status (serum iron, ferritin, transferrin saturation) causally increases AF risk, whereas higher transferrin levels are inversely correlated^[3,4]^. This paradigm shift suggests that iron dysregulation is not merely a marker but a modifiable contributor to AF pathogenesis, opening new avenues for targeted interventions.

Secondly, while the study suggests prognostic impact due to oxidative stress and inflammation, the underlying mechanisms are more intricate[5]. Excess iron promotes reactive oxygen species, lipid peroxidation, and mitochondrial dysfunction, leading to cardiomyocyte damage and arrhythmias. Iron overload can displace calcium, which can lead to altered electrical conduction and atrial fibrillation. Emerging evidence also points to ferroptosis as a contributor to AF, with experimental inhibition showing promise. This intricate network of oxidative stress, mitochondrial dysfunction, ion channelopathy, inflammation, and ferroptosis is not a collection of isolated pathological events but a synergistic interplay where each element amplifies the others, culminating in myocardial damage and arrhythmogenesis.

Thirdly, the machine learning (ML) model’s prognostic value is promising (AUC 0.71 testing). However, the single-center design limited the sample size, which affects generalizability, and potential unmeasured confounders may persist due to the retrospective nature of the data. While the authors commendably employed SHAP analysis to elucidate feature contribution and interpret the optimal model, a key challenge for complex “black box” models remains full clinician trust and adoption[6]. Future research must prioritize multi-center designs with external validation and further integrate explainable AI techniques to enhance transparency and facilitate clinical integration.

To summarize, this study undeniably highlights the crucial interplay between iron homeostasis and cardiac outcomes. Future research must address causality, refine mechanistic understanding, and enhance ML model utility through generalizability and interpretability. This collaborative, multidisciplinary effort is essential to translate scientific findings into actionable, safe clinical strategies for critically ill AF patients.

## Data Availability

All data are included in the article.
